# Antioxidant and Anti-Inflammatory Activities of the Crude Extracts of *Moringa oleifera* from Kenya and Their Correlations with Flavonoids

**DOI:** 10.3390/antiox8080296

**Published:** 2019-08-09

**Authors:** Yong-Bing Xu, Gui-Lin Chen, Ming-Quan Guo

**Affiliations:** 1 Key Laboratory of Plant Germplasm Enhancement and Specialty Agriculture, Wuhan Botanical Garden, Chinese Academy of Sciences, Wuhan 430074, China; 2 Graduate University of Chinese Academy of Sciences, Beijing 100049, China; 3 Sino-African Joint Research Center, Chinese Academy of Sciences, Wuhan 430074, China

**Keywords:** *Moringa oleifera*, antioxidant, anti-inflammatory, flavonoids, phytochemical profile

## Abstract

*Moringa oleifera* Lam. (*M. oleifera*) is commonly distributed and utilized in tropical and sub-tropical areas. There has been a large number of reports on the antioxidant and anti-inflammatory activity of its leaves, but only a few about its seeds and roots. Hence, in this work we aimed to systematically compare the antioxidant and anti-inflammatory activities of the ethanol crude extracts of leaves, seeds, and roots of *M. oleifera* from Kenya, and further correlate the differential activities with the chemical constituents from these three parts. The antioxidant activities were measured by using three different assays (DPPH (2,2-diphenyl-1-picrylhydrazyl), ABTS (2,2′-azinobis-(3-ethylbenzthiazoline-6-sulfonic acid) and FRAP (Ferric-Reducing Antioxidant Power), respectively). Results showed that the leaf extracts displayed the highest DPPH radical scavenging and FRAP total reducing power activities with IC_50_ values of 1.02 ± 0.13 mg/mL and 0.99 ± 0.06 mM Fe^2+^/g, respectively; the leaf and root extracts exhibited potential ABTS radical scavenging activities with the IC_50_ values of 1.36 ± 0.02 and 1.24 ± 0.03 mg/mL. Meanwhile, the leaf and seed extracts (11.1–100 µg/mL) also exerted obvious anti-inflammatory activities, as indicated by the inhibition of NO production. To further reveal correlations between these differential activities with the chemical constituents in the three organs, the total flavonoids content (TFC) of the three different extracts were evaluated, and the TFC of leaves, seeds and roots were found to be 192.36 ± 2.96, 5.89 ± 0.65 and 106.79 ± 2.12 mg rutin equivalent (RE)/g, respectively. These findings indicated the important impacts of the total flavonoid contents on antioxidant and anti-inflammatory activities. Additionally, we further determined the phytochemical profiles of *M. oleifera* by HPLC-UV/ESI-MS/MS, and identified most of the chemical constituents of leaves as flavonoids. In summary, the leaves of *M. oleifera* are a better potential natural source of antioxidants and anti-inflammatory agents, and very promising for development into the health promoting dietary supplements.

## 1. Introduction

*Moringa oleifera* Lam. (*M. oleifera*) is widely distributed and utilized in tropical and sub-tropical regions of the world, and is mainly native to India and Africa. It is often referred to as drumstick tree or horseradish tree, and also called “miracle tree”, or “natural gift”, or “mother’s best friend”, due to the high nutrients in leaves with protein, minerals, and β-carotene [1]. The *M. oleifera* leaves can be eaten in multiple ways, and can maintain nutrient levels for a long time when stored as dried powder. Therefore, some reginal and international relief organizations are putting a great deal of emphasis on developing the leaves of *M. oleifera* as a nutritional supplement in some Africa countries [2]. 

Traditionally, in some countries like India, Pakistan and Uganda, *M. oleifera* has long been widely used to relieve various diseases, such as diabetes, obesity, hysteria, scurvy, and even tumors [3,4]. It is reported that the *M. oleifera* contains many phytoconstituents such as flavonoids [5], alkaloids [6], saponins [7], saccharides [8], glucosinolates [9], tannins [10], phenolic acids [11], and nitrile glycosides [12], etc. These complex natural phytochemicals contribute to its numerous pharmacological activities. For example, *M. oleifera* leaves usually display good anti-inflammatory [5], anti-cancer [13], antioxidant [14], antibacterial [15], hepatoprotective [16], cardioprotective [6], anti-hypertensive [17], hypolipidemic [18], hypoglycemic [19] activities, and so on; the seeds show distinct antimicrobial [20], antidiabetic [21], anti-inflammatory [22] activities, etc.; the roots could also possess some biological activities, such as anti-inflammatory [23], anti-cancer [24], antiulcer [25], antifertility [26], antiurolithiatic [27] activity and the like. Hence, *M. oleifera* has been widely studied in recent years because of its enormous potential as a source of healthy food of medicinal value.

There has been a large amount of reports about the antioxidant and anti-inflammatory activities of *M. oleifera* leaves, but only a few referred to other parts of *M. oleifera*. For example, Ndhlala et al. [28] had determined the antioxidant activities of leaf extracts of thirteen *M. oleifera* cultivars from Thailand, South Africa and United States of America, etc. Siddhuraju et al. [29] had examined the antioxidant activities of *M. oleifera* leaves grown in three different agroclimatic regions (India, Nicaragua and Niger, respectively). Coppin et al. [5] had compared the anti-inflammatory activities of three samples of *M. oleifera* leaves from sub-Sahara Africa. In this respect, those previous reports showed the *M. oleifera* leaves are rich in flavonoids and phenolic compounds with high antioxidant and anti-inflammatory activities, whereas there is no such characterization of *M. oleifera* collected in Kenya, let alone a systematic comparison of its phytochemicals of three different organs (leaves, seeds and roots) as well as the correlation to their different biological activities.

Hence, the objective of this work was to systematically compare the antioxidant and anti-inflammatory activities of three different organs of *M. oleifera* from Kenya, and to correlate them with the chemical constituents of the organ itself. To this end, this study firstly measured the antioxidant activities of the three organs (leaves, seeds and roots) of *M. oleifera* and their total flavonoids content, and further determined their anti-inflammatory activities, and then finally evaluated their phytochemical profiles by LC-MS. We expect that the systematic comparison of three different organs of *M. oleifera* from Kenya could provide some new findings to explain or explore its traditional use or new development, and meanwhile provide valuable information on *M. oleifera* as a natural source of antioxidants and anti-inflammatory agents to promote their applications for functional food or medicinal values in the near future.

## 2. Materials and Methods

### 2.1. Plant Materials

The three different fresh parts (young leaves, seeds, and roots) of *M. oleifera* (6 years old) were collected from a farm in Machakos County (Kenya) with rich black soil and plenty of water in June of 2018. These fresh plant materials were dried in the sun, and pulverized by a disintegrator, then packed in sealed polyethylene bags and stored in a fridge at 4 °C until use. The specimens were authenticated by a botanist (Prof. Guangwan Hu) of Wuhan Botanical Garden of Chinese Academy of Sciences, and were stored in the herbarium of the Key Laboratory of Plant Germplasm Enhancement and Specialty Agriculture with the voucher specimen numbers (No. 2018001-2018003). 

### 2.2. Chemicals and Reagents

Rutin was purchased from J&K Scientific Ltd. (Beijing, China), and aspirin (ASP), chloramphenicol, streptomycin, and lipopolysaccharide (LPS) were purchased from Shanghai Aladdin Bio-Chem Technology Co., LTD (Shanghai, China). The fetal bovine serum (FBS) and Dulbecco’s Modified Eagle Medium (DMEM) were obtained from Gibco (Beijing, China). LC-MS grade water was prepared with EPED (Nanjing EPED Technology Development Co., Ltd. Nanjing, China). The acetonitrile and formic acid of HPLC grade, 2,2-diphenyl-1-picrylhydrazyl (DPPH), 2,2′-azinobis-(3-ethylbenzthiazoline-6-sulfonic acid) (ABTS), 1,3,5-tri(2-pyridyl)-2.4.6-triazine (TPTZ), and 6-hydroxy-2,5,7,8-tetramethylchroman-2-carboxylic acid (Trolox) were supplied by Sigma-Aldrich Corp. (Shanghai, China). Millipore membranes (0.22 μm) were provided by Tianjin Jinteng Experiment Equipment Co., Ltd. (Tianjin, China). All other chemicals used in this study were of analytical grade, and obtained from Sinopharm Chemical Reagent Co., Ltd. (Shanghai, China), such as ascorbic acid, methanol, sodium hydroxide (NaOH) and so on.

### 2.3. Preparation of Sample Extraction

Firstly, 5 g dried powders of three different parts of *M. oleifera* (leaves, seeds, and roots) were immersed in 40 mL 90% ethanol in a 50 mL centrifuge tube for 3 h, and then extracted in an ultrasonic bath (200 W, 40 KHz) for 30 min. Next, the extracts were centrifuged for 5 min at 5000 rpm, and then the extraction process was repeated twice more as described above. After the extraction, all the supernatants were collected and concentrated to dry residues at 45 °C using a rotary evaporator under vacuum. The dried residues were kept at 4 °C until used for further tests. For further LC-MS analysis, each dried sample was dissolved in the methanol solution, and then filtered with a 0.22 μm membrane before injection into the LC-MS system.

### 2.4. Antioxidant Assays of M. oleifera

#### 2.4.1. DPPH Assay

The DPPH free radical scavenging activities of the crude extracts from three different organs of *M. oleifera* were evaluated according to a previous method of Zhu et al. [30] with some modifications. Firstly, 10 μL of the adequate diluted samples (dissolved in methanol) or the positive control solutions (Trolox and Ascorbic acid, 31.25–1000 μM) were mixed with 190 μL of methanol solution of DPPH (0.1 mM) in a 96-well plate. After 30 min of reaction at room temperature in darkness, the absorbance of the reaction mixture was recorded at 517 nm with a multifunctional microplate reader (Tecan Infinite M200 PRO, TECAN, Männedorf, Switzerland). Meanwhile, methanol was used as a blank control in this assay, and all the extracts and controls were tested in triplicate. The percentage of inhibition of DPPH free radical was estimated and computed using the following equation:

DPPH-free radical scavenging effect (%) = [(OD_C_ − OD_S)_/OD_C_] × 100, (1)

where OD_C_ is the absorbance value of the blank control, OD_S_ is the absorbance value of the tested sample or positive control, and the IC_50_ value was acquired when the DPPH free radicals were inhibited by 50%.

#### 2.4.2. ABTS Assay

The ABTS radical scavenging assay of the three different parts extracts of *M. oleifera* was conducted using a slightly modified method reported by Zhu et al. [30]. The working ABTS radical cation (ABTS^+^) solution was prepared by mixing ABTS solution (7 mM in H_2_O) and potassium persulfate (4.9 mM in H_2_O) in equal quantities, and allowed them to react at room temperature in darkness for twelve to sixteen hours. For this assay, the ABTS^+^ solution was appropriately diluted with methanol to acquire an absorbance value of 0.700 ± 0.100 at 734 nm. Then, the mixture solution consisting of 190 μL of ABTS^+^ solution and 10 μL of appropriately diluted samples, or Vitamin C solution (31.25 to 1000 μM), was incubated in a 96-well plate for 30 min in darkness before reading its absorbance at 734 nm. Methanol was used as blank and Vitamin C as positive control. All of the extracts and controls were tested in triplicate. The IC_50_ value of ABTS assay was acquired at different concentrations; the calculation formula was identical to the DPPH radical scavenging method described in Section 2.4.1.

#### 2.4.3. FRAP Assay

The FRAP assay was done in accordance to the previous method of Benzie and Strain [31]. Firstly, the working stock solution (Fe^3+^–TPTZ solution) was composed of 20 mM FeCl_3_·6H_2_O solution, 10 mM TPTZ solution in 40 mM HCl and 300 mM acetate buffer (3.1 g C_2_H_3_NaO_2_·3H_2_O and 16 mL C_2_H_4_O_2_, pH 3.6) at a ratio of 1:1:10 (v/v/v), and then stored at 37 °C before use. Thereafter, 10 μL of appropriately diluted samples were added to 30 μL of ultrapure water followed by 260 μL of the fresh Fe^3+^–TPTZ stock solutions in a 96-well plate. After 10 min for incubation at 37 °C, the absorbance of the mixture was acquired at 593 nm by triplicate tests with a multifunctional microplate reader. The calibration curve was established by using FeSO_4_·7H_2_O (62.5, 125, 250, 500, 1000, and 2000 μM) as the standard, and the FRAP activity was expressed as mM Fe^2+^/g of sample.

### 2.5. Determinations of Total Flavonoids Content (TFC)

The TFC in the dried extracts of three organs of *M. oleifera* were measured by using the colorimetric method by Zou et al. [32] with minor modifications; rutin was used as the standard. Firstly, 60 μL of adequate diluted sample solution, 360 μL of methanol and 20 μL of a 5% NaNO_2_ solution were mixed in a 1.5 mL EP tube and shaken gently for 6 min. Next, 40 μL of 10% AlCl_3_·6H_2_O was added to stand for another 6 min, and followed by the 120 μL of 4% NaOH solution. After 15 min, the absorbance of reaction mixtures was read at 510 nm with an UV/VIS spectrophotometer (UV-1100, MAPADA, Shanghai, China). Methanol was used as the blank. The TFC of each sample was expressed as mg of rutin equivalents per gram of sample (mg RE/g sample). For each sample, this assay was repeated three times.

### 2.6. Anti-Inflammatory Activities of M. oleifera

The murine macrophage RAW264.7 cells were obtained from the American Type Culture Collection (ATCC), and cultured at 37 °C in the DMEM medium supplemented with the 10% FBS and 1% penicillin-streptomycin in a humidified incubator containing 5% CO_2_. The nitrite production was measured according to the Griess reagent protocol [33]. Briefly, the cells were transferred into the 96-well plate (4–6 × 10^4^ cells in each well) and incubated for 24 h. Then, those cells were cultured with serial dilution (11.11, 33.33, 100 μg/mL) of leaves and seeds extracts of *M. oleifera* for 2 h. Next, those cells were added with the 10 ng/mL of LPS for another 24 h. Then, 100 μL of the culture medium were mixed with the equal volume of the Griess reagent, and after 15 min of reaction, the mixture absorbance was read at 540 nm with a microplate reader. Aspirin (10.0 μg/mL) was used as the positive control.

### 2.7. HPLC-UV/ESI-MS/MS Analysis

A TSQ Quantum Access MAX mass spectrometer coupled with a Thermo Accela 600 series HPLC system (Thermo Fisher Scientific, San Jose, CA, USA) was employed for the LC-MS analysis, which was equipped with both UV detector and ESI source. The separation of *M. oleifera* extracts was acquired with a Waters Sunfire RP-C18 column (4.6 × 250 mm, 5 μm) at 25 °C. 0.1% (v/v) formic acid in ultrapure water (mobile phase A) and acetonitrile (mobile phase B) were used as mobile phases. The HPLC gradient condition was optimized as follows: 8%-30% B in 0–30 min and 30%–95% B in 30–40 min for extracts of leaves; 5%–95% B in 0–40 min for extracts of seeds; and 5%–30% B in 0–20 min and 30%–95% B in 20–30 min for that of roots; respectively. All the injection volume of samples was 10 μL, the flow rate was selected at 0.8 mL/min. The ESI-MS conditions were set as following: mass range (*m/z*) was from 150 to 1500, spray voltage was 3 kV, capillary temperature was 350 °C, vaporizer temperature was 300 °C, sheath gas (N_2_) was 40 psi, auxiliary gas (N_2_) was 10 psi, and the negative scan mode was applied. Full scan and the data-dependent mode were selected to obtain the mass spectra data. All compounds in the extracts of *M. oleifera* were tentatively identified by comparing the retention time, parent ion, and mass fragments in references or available standards.

### 2.8. Statistical Analysis

In this work, all tests were done in triplicate and the data were represented as the mean ± standard deviation (SD). The statistical software SPSS 17.0 (IBM Corp., New York, NY, USA) was applied for statistical analysis.

## 3. Results and Discussion

### 3.1. Antioxidant Activity of M. oleifera Leaves, Seeds and Roots

Because of the complexity of natural phytochemicals and their different modes of action, it is inaccurate to assess the overall antioxidant potential only by a single method. Therefore, in this work we used three different methods (DPPH, ABTS, and FRAP assay, respectively) to assess and compare the antioxidant potential of three organs of *M. oleifera* [34]. The results are shown in Table 1. It was interesting to find that the leaves, seeds and roots of *M. oleifera* had the IC_50_ values of 1.02 ± 0.13, > 64, 3.33 ± 0.11 mg/mL with DPPH radical scavenging activities, whereas the ABTS values were 1.36 ± 0.02, 40.35 ± 1.47 and 1.24 ± 0.03 mg/mL, and the FRAP values were 0.99 ± 0.06, 0.02 ± 0.00 and 0.20 ± 0.01 mM Fe^2+^/g, respectively. The results of DPPH and FRAP methods were indicated that the antioxidant activities of *M. oleifera* leaves were the highest, the antioxidant activities of seeds were the lowest, and the roots were in-between. Meanwhile, the roots of *M. oleifera* had the highest potential antioxidant activities in ABTS assay. Part of these results was similar with those reported by Atawodi et al. [35], but with different activities due to different species, different organs, or different geographic regions. In their study, the descending order of antioxidant activities was root barks > leaves > stem of *M. oleifera* in Nigeria. However, they did not test the seeds.

### 3.2. Total Flavonoids Content (TFC)

The TFC of the dried extracts of *M. oleifera* leaves, seeds and roots was listed in Table 1. As shown in Table 1, the TFC of leaves, seeds and roots were 192.36 ± 2.96, 5.89 ± 0.65 and 106.79 ± 2.12 mg rutin equivalent (RE)/g, respectively. It was obvious to see that the descending order of the TFC of plant parts was: leaf > root > seed (32.66:18.13:1). As known from Table 1, the antioxidant activity of leaves is the highest, followed by the roots and the seeds. These indicated the positive impacts of the total flavonoid contents on antioxidant activities. In a previous study, the descending order of the TFC of the ethanol extracts of *M. oleifera* in Nigeria was: root > bark > leaf > flower > seed [35]. Compared with this study, the seeds from both Kenya and Nigeria had the lowest TFC, while the TFC of leaves was higher than roots in Kenya and the opposite occurred in Nigeria. The differences may be caused by the differences in climate and soil compositions of the different growing areas [36,37].

### 3.3. Anti-Inflammatory Activity

**Figure 1:**
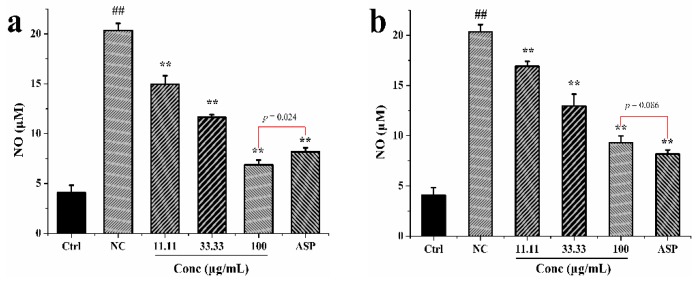
Anti-inflammatory activities of leaves (**a**) and seeds (**b**) of *M. oleifera* on NO production in RAW264.7 cells. ** *p* < 0.01, compared with the NC (negative control) group.

The anti-inflammatory activity was evaluated by determining the production of the nitric oxide (NO) in RAW 264.7 macrophage cells. As shown in Figure 1, the NO in the leaf and seed extracts treated groups was significantly lower than that of the NC group in a dose-dependent manner, within the concentration range investigated (11.11, 33.33, and 100 μg/mL). The leaf extracts at the concentration 100 μg/mL, gave lower NO production than the positive control aspirin (*p* = 0.024). In contrast, the seed extracts at the same concentration resulted in higher NO production than aspirin, but without significant difference (*p* = 0.086). Thus, it appeared that the leaf extracts of *M. oleifera* had a better anti-inflammatory potential than the seeds.

### 3.4. Analysis of M. oleifera Leaves, Seeds and Roots by HPLC-UV/ESI-MS/MS

**Figure 2:**
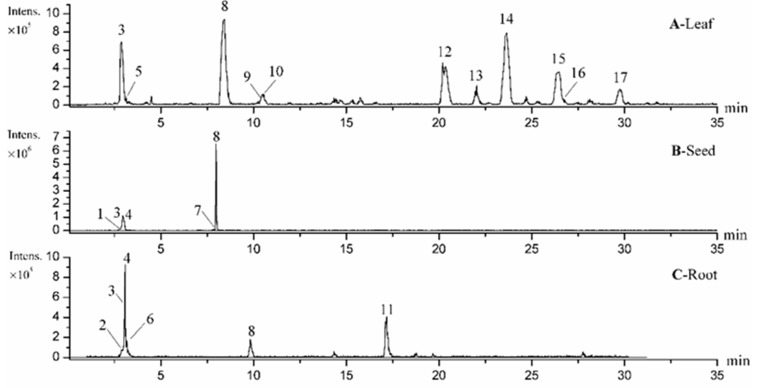
The base peak chromatogram (BPC) of *M. oleifera* leaves (**A**), seeds (**B**) and roots (**C**) extracts by LC-MS analysis in the negative ion mode.

**Figure 3:**
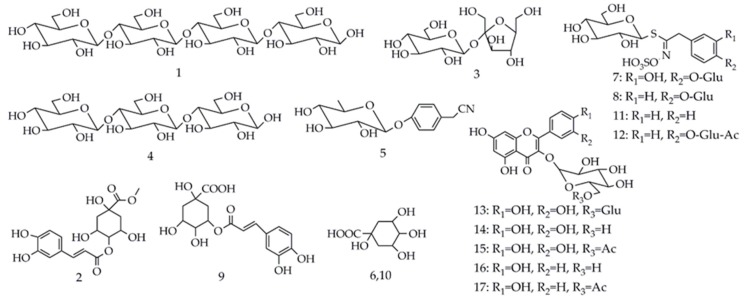
The molecular structures of all detected compounds in extracts of leaves, seeds and roots of *M. oleifera*. Glu: Glucoside; Ac: Acetyl.

Although the TFCs of three different parts of *M. oleifera* were determined and compared in our study, the phytochemical profiles or flavonoids profiles have not yet been revealed or compared so far. To further explore and explain the chemical differences in these three organs, LC-MS/MS analysis was conducted. As a result, the base peak chromatograms (BPC) of the extracts of *M. oleifera* leaves, seeds and roots with the LC-MS analysis were shown in Figure 2, where the peaks were numbered orderly according to their retention time. As per the analysis, a total of 17 compounds in three organs of the *M. oleifera* were identified or tentatively characterized in the ESI negative ion mode after comparing with the data from the references or purified standards. Table 2 summarized mass spectra data of all the compounds in extracts, which included saccharides, nitrile glycosides, organic acids, phenolic acids, phenylpropanoids, glucosinolates and flavonoids, and their chemical structures were presented in Figure 3. As shown in Figure 2 and Table 2, there were two shared constituents of peak **3** and peak **8** identified in all the three organs of *M. oleifera*. However, there were obvious differences in the metabolite profiles of the extracts from leaves, seeds and roots of *M. oleifera*, which could provide a good clue to explain their different antioxidant power, and the main phytochemicals responsible for it. It is also found the *M. oleifera* leaves are rich in flavonoids which may contribute largely to their better antioxidant and anti-inflammatory activities, while the seeds are not. In this sense, the present study for the first time conducted the phytochemical comparisons of three different organs of *M. oleifera* (leaves, seeds and roots) which could have a good correlation to their distinct antioxidant or anti-inflammatory activities, as shown in Table 1 and Figure 1.

#### 3.4.1. Saccharides and Nitrile Glycosides

As shown in Table 2, peak **1** had a negative molecular ion [M−H]^−^ at *m/z* 665 and peak **4** had a molecular ion [M−H]^−^ at *m/z* 503; according to the report by Peng et al. [38], they could be proposed as cellotetraose and cellotriose. Owing to the same product ions at *m/z* 179, 119, 113, 101, and 89, the molecular ion [M−H]^−^ for peak **3** at *m/z* 341 was identified as sucrose by comparing their mass spectra data with the previous literature [39]. Peak 5 showed the molecular ion [M−H]^−^ at *m/z* 278. Its main product ions at *m/z* 188 and 158 correspond to the [M−H−90]^−^ and [M−H−120]^−^, which are the characteristic diagnostic ions of glycone [40]. Besides, the fragment ion at *m/z* 116 was generated by the loss of the hexose moiety ([M−H−162]^−^). Hence, peak **5** was temporarily proposed as niazirin [41].

#### 3.4.2. Organic Acids and Phenylpropanoids

Both peaks **6** and **10** exhibited the same molecular ion [M−H]^−^ at *m/z* 191 with the same fragment ions at *m/z* 173, 127 and 85, suggesting that they were structural isomers. In addition, due to the product ions at *m/z* 173 ([M−H−18]^−^, loss of a H_2_O) and at *m/z* 127 ([M−H−64]^−^, loss of a H_2_O and HCOOH), peak **6** and **10** were identified as quinic acid isomer 1 and 2, respectively [42]. Peak **2** showed the molecular ion [M−H]^−^ at *m/z* 367 and its product ions at *m/z* 277 ([M−H−90]^−^) and 205 ([M−H−162]^−^), indicating the presence of a glycoside for peak **2**. Thus, peak **2** was tentatively identified as methyl 4-caffeoylquinate [41].

#### 3.4.3. Glucosinolates

By referring to the previously published report [9], four glucosinolates (peak **7**, **8**, **11**, **12**) were identified as 3-hydroxy-4-(α-l-rhamnopyranosyloxy) benzyl glucosinolate (peak **7**), glucomoringin (peak **8**), glucotropaeolin (peak **11**) and acetyl-4-(α-l-rhamnopyranosyloxy) benzyl glucosinolate (peak **12**), respectively. All of them shared common characteristic fragments, *m/z* 97 and 259. The specific characteristic ion at *m/z* 97 was assigned as the [SO_4_H]^−^ ion, and the major ion peak at *m/z* 259 ([SO_4_H+162]^−^) was deduced as being sulfated glucose moiety.

#### 3.4.4. Phenolic Acids and Flavonoids

Peak **9** was tentatively identified as 3-caffeoylquinic acid according to the MS/MS data reported by Clifford et al. [43]. In this study, 5 peaks (peak **13**, **14**, **15**, **16** and **17**) were identified as flavonoids. For peak **13**, compared with the mass spectra data of rutin standard under the same condition of LC-MS, it was definitely identified as rutin. Peak **14** and **15** showed common product ions at *m/z* 301 and 300 due to the quercetin core, and *m/z* 463 corresponded to quercetin glycoside. Similarly, peak **16** and **17** shared common product ions at *m/z* 285 and 284 due to the kaempferol core, and *m/z* 447 corresponded to kaempferol glycoside. By comparing with the mass spectra data in previously reported literature, peak **14–17** were tentatively identified as quercetin 3-*O*-glucoside, quercetin-acetyl-glycoside, kaempferol 3-*O*-glucoside and kaempferol-acetyl-glycoside [2], respectively. In this study, there were no flavonoids identified in root of *M. oleifera*, but in the TFC assay, the TFC of root of *M. oleifera* was 106.79 ± 2.12 mg rutin equivalent (RE)/g, and the reason for this may be due to the presence of ortho-diphenolic hydroxyl analogues, such as quinic acid in the roots, since the ortho-diphenolic hydroxyl compounds can coordinate with aluminum ions, and thus influence the determination of TFC.

## 4. Conclusions

In this study, the antioxidant assays with DPPH, ABTS and FRAP, anti-inflammatory activity tests by means of the effects on the NO production, and phytochemical analysis with HPLC-UV/ESI-MS/MS were conducted to compare and correlate the antioxidant and anti-inflammatory activities with chemical compositions among the leaves, seeds and roots of *M. oleifera* in Kenya. To the best of our knowledge, this is the first study aiming to reveal the correlations between the phytochemical profiles and antioxidant and anti-inflammatory activities of three different organs of *M. oleifera* from Kenya. As a result, we revealed that leaves of *M. oleifera* are rich in flavonoids and phenolic acids, which explained the best potential antioxidant and anti-inflammatory activities among three organs. This current study revealed the positive correlation between the flavonoids content in three different organs of *M. oleifera* with their antioxidant and anti-inflammatory activities, which suggested that the leaves from *M. oleifera* could be a more potential natural source of antioxidants and anti-inflammatory source than that of other organs, and is very promising for development into health promoting dietary supplements now and in the near future.

## Figures and Tables

**Table 1:** Total flavonoids content and their corresponding antioxidant activity of *M. oleifera*.

Sample	IC_50_ of DPPH * (mg/mL)	IC_50_ of ABTS * (mg/mL)	FRAP * (mM Fe^2+^/g)	TFC * (mg RE/g)
Leaves	1.87 ± 0.03 ^c^	1.36 ± 0.02 ^b^	0.99 ± 0.06 ^a^	192.36 ± 2.96 ^a^
Seeds	>64 ^a^	40.35 ± 1.47 ^a^	0.02 ± 0.00 ^c^	5.89 ± 0.65 ^c^
Roots	3.33 ± 0.11 ^b^	1.24 ± 0.03 ^b^	0.20 ± 0.01 ^b^	106.79 ± 2.12 ^b^
Trolox	0.10 ± 0.01 ^d^	NT *	NT	NT
Ascorbic acid	0.05 ± 0.00 ^e^	0.11 ± 0.01 ^b^	NT	NT

* All of the data were expressed as means ± standard deviation; DPPH: 2,2-diphenyl-1-picrylhydrazyl; ABTS: 2,2′-azinobis-(3-ethylbenzthiazoline-6-sulfonic acid); FRAP: Ferric-Reducing Antioxidant Power; TFC: Total flavonoids content; Half maximal inhibitory concentration (IC_50_) value was acquired when DPPH and ABTS radicals were inhibited by 50%; The FRAP value was represented as mM Fe^2+^/g of sample; Mean values with different letters (^a–e^) within a row were significantly different at a level of *p* < 0.05 by DMRT (Duncan’s multiple range test); NT: Not tested.

**Table 2:** Compounds identified from leaves, seeds and roots of *M. oleifera* by LC-MS/MS.

Peak No. ^a^	RT ^b^ (min)	[M−H]^−^	Molecule Formula	MS^2^ (*m/z*)	Identification	Part ^c^	Reference
Saccharides							
1	2.83	665	C_24_H_42_O_21_	485, 383, 341, 179	Cellotetraose	S	[38]
3	2.88	341	C_12_H_22_O_11_	179, 161, 131, 119, 113, 101, 89	Sucrose	L, S, R	[39]
4	2.90	503	C_18_H_32_O_16_	383, 323, 281, 221, 179, 119, 89	Cellotriose	S, R	[38]
Phenylpropanoids							
2	2.87	367	C_17_H_20_O_9_	277, 205, 187, 157, 113	Methyl 4-caffeoylquinate	R	[41]
Nitrile Glycosides							
5	2.95	278	C_14_H_17_NO_5_	212, 188, 158, 116, 101	Niazirin	L	[41]
Organic acids							
6	3.20	191	C_7_H_12_O_6_	173, 127, 111, 85	Quinic acid isomer 1	R	[42]
10	10.55	191	C_7_H_12_O_6_	173, 127, 87, 85	Quinic acid isomer 2	L	[42]
Glucosinolates							
7	7.94	586	C_20_H_29_NO_15_S_2_	440, 390, 344, 259, 198, 164, 97	3-Hydroxy-4-(α-l-rhamnopyranosyloxy) benzyl glucosinolate	S	[9]
8	8.44	570	C_20_H_29_NO_14_S_2_	490, 328, 275, 259, 241, 97, 96	Glucomoringin	L, S, R	[9]
11	16.90	408	C_14_H_19_NO_9_S_2_	259, 241, 215, 212, 195, 166, 97	Glucotropaeolin	R	[9]
12	20.20	612	C_22_H_31_NO_15_S_2_	370, 275, 259, 241, 106, 97	Acetyl-4-(α-l-rhamnopyranosyloxy) benzyl glucosinolate	L	[9]
Phenolic acids							
9	10.44	353	C_16_H_18_O_9_	191, 179, 135	3-Caffeoylquinic acid	L	[43]
Flavonoids							
13	22.03	609	C_27_H_30_O_16_	301, 300, 271	Rutin	L	standard
14	23.65	463	C_21_H_20_O_12_	301, 300, 271	Quercetin 3-*O*-glucoside	L	[2]
15	26.47	505	C_23_H_22_O_13_	301, 300, 271	Quercetin-acetyl-glycoside	L	[2]
16	26.61	447	C_21_H_20_O_11_	285, 284, 255	Kaempferol 3-*O*-glucoside	L	[2]
17	29.75	489	C_23_H_22_O_12_	285, 284, 255	Kaempferol-acetyl-glycoside	L	[2]

^a^ Peak number was ordered according to the retention time; ^b^ RT: retention time; ^c^ Part including L: leaf, S: seed, R: root.
